# Predictors of caesarean section in Northern Ghana: a case-control study

**DOI:** 10.11604/pamj.2018.29.20.13917

**Published:** 2018-01-19

**Authors:** Paschal Awingura Apanga, John Koku Awoonor-Williams

**Affiliations:** 1 Talensi District Hospital, Ghana Health Service, Tongo, Ghana; 2 Policy, Planning, Monitoring and Evaluation Division, Ghana Health Service, Accra, Ghana

**Keywords:** Caesarean section, case-control, predictor, risk factor, retrospective

## Abstract

**Introduction:**

Caesarean section rates have become a global public health. This study investigated obstetric and socio-demographic factors associated with caesarean section in northern Ghana.

**Methods:**

This was a case-control study comparing 150 women who had caesarean section (cases) and 300 women who had vaginal delivery (controls). Data were collected retrospectively from delivery registers, postpartum and postnatal registers in the Bolgatanga Regional Hospital. Univariate and multivariate analysis of data were done using SPSS 22.

**Results:**

The study revealed that women who had higher odds of having a caesarean section were women who; attended Antenatal care (ANC) ≥ 4 times (Adjusted OR= 2.99, 95% CI1.762-5.065), were referred from other health facilities (Adjusted OR = 1.19, 95% CI 1.108-1.337) and had a foetal weight of ≥ 4000 grams (Adjusted OR = 1.21, 95% CI 1.064-1.657). There was a slight increase in odds of having a caesarean section among women who had a gestational age > 40 weeks (Adjusted OR = 1.09, 95% CI 1.029-1.281). Women who had secondary/higher education (Adjusted OR = 0.55, 95% CI 0.320-0.941), gestational age < 37 weeks (Adjusted OR = 0.20, 95% CI: 0.100-0.412) and women who had a foetal weight of 1500 grams to 2499 grams (Adjusted OR = 0.17, 95% CI 0.086-0.339) were associated with a lower odds of having a caesarean section.

**Conclusion:**

There was an increase in odds of having a caesarean section among pregnant women who had a foetal weight of ≥ 4000 grams and women who attended ANC ≥ 4 times. Pregnant women who were referred also had increase odds of having a caesarean section.

## Introduction

Caesarean Section (CS) is a surgical obstetric care that is beneficial in saving lives of mothers and newborns when its indication is well grounded [1-3]. The decision to perform a CS should be based on obstetric history and anticipated mode of delivery [4]. Clients should be provided with detailed information on CS during prenatal care. This ensures that clients are well informed when CS is indicated. In Nigeria it was found that higher perinatal mortality of 34% was associated with clients who refused elective CS as compared to 5% of clients who accepted the procedure [5]. The refusal for CS could be attributed to lack of detailed information on CS among clients. Caesarean section is not only a life saving intervention but its rates have been used as an indicator of access to emergency obstetric care in population based studies [6, 7]. However, like any other surgery, CS carries the risk of complications including death [8]. Caesarean section rates have become a global public health concern as the World Health Organization (WHO) observed that some countries have unacceptable high CS rates above the recommended rate of 15% [9]. Caesarean section rates have been reported to be very high. The rise in CS rates has been attributed to many factors. Nulliparity, grand multiparity, macrosomia, hypertensive disorders in pregnancy, extremes of maternal age and cephalopelvic disproportion have been observed as indications for rise in CS rates [10-13]. Others indications documented include previous CS, antepartum haemorrhage, multiple pregnancy, maternal height, maternal weight, preference by clients and private healthcare have also been responsible for high CS rates globally [14-17]. However, increased CS rates have also been attributed to medically unjustifiable indications which makes it more alarming, hence bringing negative economic and health related repercussions [1]. This is of great public health concern as indiscriminate use of this procedure may put the health of mothers and newborns at risk. In Sub-Saharan Africa, countries like Ghana have a health system that is structured with most of the deliveries initiated in health facilities that do not have the capacity to perform CS and lack ambulances for referrals of clients who need CS [7, 15]. This often leads to a lot of pregnant women undergoing emergency CS at referral hospitals with adverse obstetric outcomes as compared to clients who have been booked for parturient [15]. Although the risk factors of caesarean section have been documented globally [11, 18], not much has been done to assess the predictors of CS locally within a specific Ghanaian population. This study therefore sought to determine the predictors of caesarean section. This we believe is essential for prenatal counselling in Ghana.

## Methods

**Figure 1:**
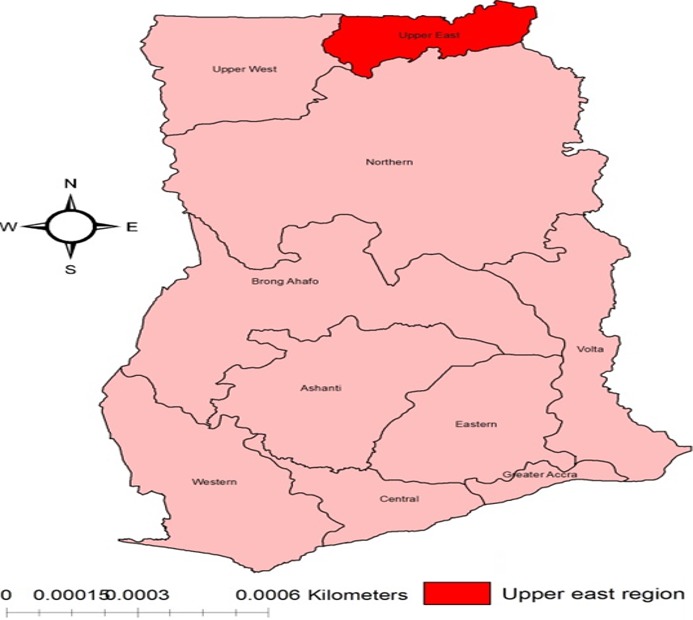
The map of Ghana showing the upper East region

**Study design, setting and participants**: This is a case control study. Records of Ghana Health Service (GHS) delivery registers, postpartum and postnatal registers in the obstetrics and gynaecology department of the regional hospital were reviewed from 1^st^ January, 2015 to 31^st^ May, 2015. Records of pregnant women who reportedly delivered in the hospital were reviewed. Participants were recruited on the basis of cases and controls. Cases were defined as mothers who had caesarean section (elective or emergency caesarean section) within the review period whilst the control group were mothers who had vaginal delivery (spontaneous or assisted vaginal delivery) within same review period. Mothers who had multiple gestation and incomplete information were excluded from the review. The study was conducted in the Bolgatanga Regional hospital located in the Upper East region (Figure 1) in northern Ghana which shares boundaries with Bongo District to the North, to the East with Nabdam District, to the South with Talensi District and to the West with Kassena-Nankana Municipality [19]. The Bolgatanga Regional hospital is the main referral facility in the region and provides healthcare services to the entire population of the region [19, 20].

**Sample size and data collection procedures**: Sample size was calculated using Epi info 7 with a 95% two sided confidence interval, power of 80%, two controls per case based on the formula by Kelsey et al [21]. We assumed that 15% (recommended WHO accepted CS rate) of pregnant women who delivered at the Bolgatanga Regional hospital were likely to have a CS, this was used in the calculation of the sample size [9]. Using Epi info StatCalc, our sample size was fixed at 150 cases and 300 controls (one case to two controls). The first case selected was followed by the next two successive controls as it was possible to have two successive cases. This was repeated until the sample size was obtained. Records review guide was employed in selecting cases and controls during the review of records. Data were collected by researchers by reviewing the GHS delivery registers, postpartum and postnatal registers in the obstetrics and gynaecological department of the Bolgatanga Regional hospital. The data were securely stored and entered into SPSS version 22 in a computer which was encrypted and password protected. The records review guide contained nine (9) variables (mode of delivery, age, educational level, parity, gestational age, antenatal care attendance, foetal weight, HIV status and referral status) which were developed in line with obstetric and socio-demographic characteristics of clients contained in the GHS delivery registers, postpartum and postnatal registers.

**Data analysis**: Data were entered into SPSS version 22 and cleaned before analysis. The mean, standard deviation and percentages were used for descriptive analysis of obstetric and socio-demographic characteristics of clients. Univariate logistic regression analysis was used to compare the associations between the outcome variable (caesarean section) and independent variables such as age, educational level, parity, gestational age, antenatal care attendance, foetal weight, HIV status and referral status of clients, using the Odd Ratio (OR) and 95% Confidence Interval (CI). The likelihood ratio was used in estimating the OR and 95% CI of the associations of interest. Multivariate logistic regression was performed to examine the simultaneous effects of multiple factors whilst controlling the effects of confounding factors. To form the best fitting model which is parsimonious but biologically sound, variables with p < 0.25 from the Univariate analysis [22], variables that are clinically important to caesarean section as well as variables that had a significant association with caesarean section were all included into the multivariate model. Wald test was used in assessing the significance of the interaction of each variable included in the model. The maximum likelihood ratio was also used in estimating the parameters of the logistic regression model. The adjusted ORs and 95% CI were computed using estimates of parameters included in the final model. The Probability (P) value of less than 0.05 was considered statistically significant. The fitness of the model was assessed using the Hosmer-Lemeshow's goodness-of-fit test, classification table and area under receiving operating characteristics curve [22, 23]. The null hypothesis for Hosmer-Lemeshow's goodness-of-fit test of the model was fit [24]. The P value was 0.529 which is not significant, hence the model was fit. The classification table by SPSS also showed that 79.9% of the cases were predicted accurately irrespective of whether they had caesarean section or not. This is a good model as its predictive value is 70% and above [22], the model was more appropriate as none of the interactions were significant and there were no multicollinearity problems.

## Results

A total of 450 subjects (150 cases and 300 controls) were enrolled in the study. The mean age of subjects in the study was 27.5 with Standard Deviation (SD) of ± 6.9. The age of subjects reviewed ranged from 14 to 47 years. Table 1 shows obstetric and socio-demographic characteristics of the study population. The Univariate logistic regression results showed that pregnant women who delivered in the Bolgatanga Regional hospital, who had gestational age greater than 40 weeks (OR = 1.05, 95% CI 1.018-1.136), attended antenatal care for 4 or more times (OR% = 2.71, 95% CI 1.797-4.093), were referred to the regional hospital (OR = 1.16, 95% CI 1.103-1.257) and who had a foetal birth weight of 4000 grams or more (OR = 1.19, 95% CI 1.071-1.499) were more likely to undergo a caesarean section. Pregnant women who had a secondary/higher education (OR = 0.63, 95% CI 0.412-0.975), gestational age less than 37 weeks (OR = 0.12, 95% CI 0.068-0.225) and who had a foetal birth weight less than 2500 grams (OR = 0.13, 95% CI 0.077-0.233) were less likely to undergo a caesarean section. This is illustrated in Table 2. Variables with P value < 0.25 [22], variables of known clinical importance and variables that were statistically significant at univariate model were included in the multivariate model. Variables that were included are; educational level, gestational age, antenatal care (ANC) attendance, referral status and birth weight. The multivariate logistic regression model revealed that clients who delivered at the hospital, who had a secondary/higher education had lower odds of having a CS by 45% than clients who had no/primary education (Adjusted OR = 0.55, 95% CI 0.320-0.941). Clients who had a gestation of less than 37 weeks had a lower odds of having a CS by 80% than clients who had a gestation of 37-40 weeks (Adjusted OR = 0.20, 95% CI: 0.100-0.412). Also, clients who had a foetal weight of 1500 grams to 2499 grams had a lower odds of having a CS by 83% than clients who had a foetal weight of 2500 grams to 3999 grams (Adjusted OR = 0.17, 95% CI 0.086-0.339). However, factors associated with higher odds of having a CS were; clients who had a gestational age greater than 40 weeks (Adjusted OR = 1.09, 95% CI 1.029-1.281), clients who attended ANC for 4 or more times (Adjusted OR = 2.99, 95% CI1.762-5.065), clients who were referred from other health facilities (Adjusted OR= 1.19, 95% CI 1.108-1.337) and clients who had a foetal weight of 4000 grams and above (Adjusted OR = 1.21, 95% CI 1.064-1.657). The multivariate model is shown in Table 3.

**Table 1:** Obstetric and socio-demographic characteristics of study population

	Caesarean section		
Variable	Yes	No	Total
	n=150	n=300	n=450
	n (%)	n (%)	n (%)
**Age group**			
20-29	75 (50.0)	157 (52.3)	232 (51.6)
< 20	16 (10.7)	38 (12.7)	54 (12.0)
30-39	51 (34.0)	91 (30.3)	142 (31.6)
≥ 40	8 (5.3)	14 (4.7)	22 (4.9)
**Education**			
No/primary education	42 (28.0)	113 (37.7)	155 (34.4)
Sec/higher education	102 (68.0)	174 (58.0)	276 (61.3)
Not available	6 (4.0)	13 (4.3)	19 (4.2)
**HIV status**			
Negative	138 (92.0)	255 (85.0)	393 (87.3)
Positive	5 (3.3)	10 (3.3)	15 (3.3)
Not available	7 (4.7)	35 (11.7)	42 (9.3)
Parity			
0	31 (20.7)	55 (18.3)	86 (19.1)
0-4	99 (66.0)	200 (66.7)	299 (66.4)
≥ 5	15 (10.0)	36 (12.0)	51 (11.3)
Not available	5 (3.3)	9 (3.0)	14 (3.1)
**Gestational age**			
37-40	92(61.3)	243 (81.0)	335 (74.4)
< 37	6 (4.0)	40 (13.3)	46 (10.2)
> 40	52 (34.7)	17 (5.7)	69 (15.3)
**ANC attendance**			
0	2 (1.3)	15 (5.0)	17 (3.8)
1-3	78 (52.0)	83 (27.7)	161 (35.8)
≥ 4	70 (46.7))	202 (67.3)	272 (60.4)
**Referral status**			
Not referred	74 (49.3)	257 (85.7)	331 (73.6)
Referred	76 (50.7)	43 (14.3)	119 (26.4)
**Birth weight(grams)**			
2500-3999	87 (58.0)	259 (86.3)	346 (76.9)
< 1500	0 (0.0)	2 (0.7)	2 (0.4)
1500-2499	5 (5.3)	17 (5.7)	25 (5.6)
≥ 4000			
	55 (36.7)	22 (7.3)	77 (17.1)

ANC = Antenatal care, Refereed = clients sent by other health facilities to deliver at the regional hospital

Not Referred = Clients by their own decision who walked into the regional hospital to deliver

**Table 2:** Univariate logistic analysis of factors associated with caesarean section delivery

Variable	OR	95%CI	P
**Age group**			
20-29	^+^1	-	-
< 20	0.84	0.336-2.079	0.700
30-39	0.74	0.259-2.099	0.567
≥ 40	0.98	0.385-2.495	0.967
**Education**			
No/primary education	^+^1	-	-
Sec/higher education	0.63	0.412-0.975	0.038^++^
**HIV status**			
Negative	^+^1	-	-
Positive	1.08	0.363-3.230	0.887
**Parity**			
0	^+^1	-	-
0-4	1.34	0.642-2.852	0.427
≥ 5	1.19	0.621-2.273	0.603
**Gestational age (weeks)**			
37-40	^+^1	-	-
< 37	0.12	0.068-0.225	<0.001^++^
> 40	1.05	1.018-1.136	<0.001^++^
**ANC attendance**			
0	^+^1	-	-
1-3	0.39	0.086-1.725	0.212
≥ 4	2.71	1.797-4.093	<0.001^++^
**Referral status**			
Not referred	^+^1	-	-
Referred	1.16	1.103-1.257	<0.001^++^
**Birth weight (grams)**			
2500-3999	^+^1	-	-
1500-2499	0.13	0.077-0.233	<0.001^++^
≥ 4000	1.19	1.071-1.499	0.001^++^

Results by Univariate analyses; OR = Odd Ratios; CI = Confidence Interval; p = probability value; ^+^Reference

Category, ^++^significance at 0.05 level

**Table 3:** Multivariate logistic analysis of factors associated with caesarean section delivery

Variable	OR	95%CI	P
Education			
No/primary education	^+^1	-	-
Sec/higher education	0.55	0.320-0.941	0.029^++^
Gestational age			
37-40	^+^1	-	-
<37	0.20	0.100-0.412	<0.001^++^
>40	1.09	1.029-1.281	<0.001^++^
ANC attendance			
0	^+^1		
1-3	0.30	0.059-1.535	0.418
≥4	2.99	1.762-5.065	<0.001^++^
Referral status			
Not referred	^+^1	-	-
Referred	1.19	1.108-1.337	<0.001^++^
Birth weight (grams)			
2500-3999	^+^1	-	-
1500-2499	0.17	0.086-0.339	<0.001^++^
≥4000	1.21	1.064-1.657	0.008^++^

Results by multivariate analyses; OR = Odd Ratios; CI = Confidence Interval; p = probability value; ^+^Reference

Category, ^++^significance at 0.05 level, model fits reasonably well. Model assumptions are met. There are no interaction and multicollinearity problems

## Discussion

Our results are consistent with findings of Sørbye et al and Nilsen et al in Tanzania who reported that pregnant women who were referred were more likely to have a CS during delivery than pregnant women who walked in for delivery [7, 15]. This is because pregnant women who are usually referred from other health facilities often require emergency caesarean section as referral facilities often lack the capacity [15]. This finding suggest that health facilities that lack CS services should refer pregnant women in labour with complications early enough as they are most likely to undergo CS. Macrosomia (foetal weight ≥ 4000 grams) as defined by Oluwarotimi et al was found in this study as an independent predictor of CS. This finding agrees with many other studies that identified macrosomia as a risk factor for CS [25, 26] This finding is important as it would guide healthcare workers to take a timely decision of conducting a CS when a pregnant woman in labour presents with macrosomia. The findings also revealed that pregnant women with a gestational age of greater than 40 weeks were at a higher risk of having a CS. This finding is in line with results reported in studies by Osava et al in South eastern Brazil [25]. This is likely so because pregnant women with such gestational age category are more likely to have post term pregnancy which tend to be associated with higher risk of having a CS [27]. This finding is significant as it will inform healthcare workers on the need to carry out CS for pregnant women who have exceeded their expected date of delivery without having a spontaneous or induced labour. In addition, pregnant women who attended ANC 4 or more times were found to be more likely to have a CS. This is in contrast to findings of Tebeu et al in Cameroon who found no association between ANC attendance and CS [4]. This finding in our study may be due to pregnant women who were booked for elective CS as they are more likely to have their mode of delivery planned during ANC [28]. Such women often have medical or obstetric indications for CS and are often told by healthcare providers to report for ANC more frequently than their counterparts without such indications. In Italy, it was found that pregnant women with secondary/higher education had higher odds of having a CS than clients with no/primary education [29]. This is not in conformity with findings reported in our study as pregnant women with secondary/higher education had lower odds of having a CS than their counterparts with lower education. However, our findings were in line with studies reported by Tollånes et al in Norway who argue that pregnant women with lower education had a higher risk of having a CS as they may not be well informed about their health and often report late to health facilities with complications making them more prone to CS [30].

This study also found that pregnant women with gestational age of less than 37 weeks have lower odds of having a CS as compared to their counterparts with gestational age of 37 weeks to 40 weeks. This is likely so because pregnant women with gestational age of less than 37 weeks who go into labour usually have preterm labour [31]. Preterm labour tends to be associated with low birth weight (less than 2500 grams) [32], both of which are associated with lower risk of CS [33]. In addition, low foetal birth weight in this study is associated with lower odds of having a CS as compared to neonates with normal birth weight (2500-3999 grams). This finding re-emphasis the observation made by Puliyath in the Middle East who reported that low birth weight was at a lower risk factor for CSs. In the Univariate analysis, the age group of pregnant women had no statistical association with having a CS. This is in contrast to previous studies that found extremes of age to be associated with CS [34, 35]. However, our findings of no association between HIV status of pregnant women and CS agrees with studies by Calvert and Ronsmans that found no association between HIV and CS [36]. The lack of an association between HIV and CS in our study is surprising. This is because CS has been recommended for Prevention of Mother-To-Child Transmission (PMTCT) of HIV. This might be as a result of the low prevalence of HIV in our study. The evaluation of only obstetric and socio-demographic characteristics/variables of client records in the GHS delivery, postpartum and postnatal registers was a limitation as other possible predictors/risk factors were not assessed. The study did not explore medical as well as other obstetric indications for CS and reasons for referrals that could have made our understanding of predictors of CS much better. This is because they could have been underlying medical reasons for CS indication and not necessarily as a result of the variables assessed. The study was also subject to information bias as it is likely that not all information on mothers who delivered were recorded in the standard registers. The study also suffered from selection bias as it is likely that some women who could have benefited from caesarean section had assisted vaginal delivery resulting in the misclassification of an exposure like foetal weight. Although the sample size of the study was large, the findings may not be generalisable to the entire region as the data was collected over a short period (a five month period).

## Conclusion

This study found that pregnant women who were referred, pregnant women who attended ANC for four or more times, pregnant women with gestational age of greater than 40 weeks and pregnant women who had a foetal of weight of 4000 grams or more had a higher likelihood of delivering by CS. It will help improve on early referral of pregnant women by health facilities that lack the capacity of performing CS when it is indicated.

### What is known about this topic

- Pregnant women who have a gestational age of greater than 40 weeks are more likely to have a caesarean section;
- Pregnant women who have a foetal weight of equal to or greater than 4000grams are more likely to have a caesarean section.

### What this study adds

- Pregnant women who are referred from health facilities are more likely to have a caesarean section;
- Pregnant women with a secondary or higher school education are less likely to have a caesarean section.

## Competing interests

The authors declare no competing interests.
